# Depth of Anaesthesia Associated with 2.5% Isoflurane During Cardiopulmonary Bypass

**DOI:** 10.1186/1749-8090-10-S1-A135

**Published:** 2015-12-16

**Authors:** Michael D Connelly, R Peter Alston

**Affiliations:** 1 University of Edinburgh, Edinburgh, Scotland, EH16 4TJ, UK; 2 Department of Anesthesia, Critical Care and Pain Medicine, Edinburgh Royal infirmary, Edinburgh, Scotland, EH16 4SA, UK

## Background/Introduction

Since its inception, and to this day, measurement of the depth anaesthesia during cardiopulmonary bypass (CPB) has been challenging. Bispectral index (BIS) is commonly used but remains controversial. One pragmatic approach to anaesthesia is to administer 2.5% isoflurane into the sweep gas supply to the oxygenator and allow neuromuscular blockade to fade so that patient movement indicates an insufficient depth of anaesthesia.

## Aims/Objectives

BIS was used to assess the depth of anaesthesia produced when 2.5% isoflurane was administered via the oxygenator during CPB. The aim was to discover whether this dose produced a BIS score lower than the recommended range of 40-60 and therefore deemed to be excessive. Secondary aims investigated the effects of age, patient temperature and oxygenator exhaust gas isoflurane concentration on patients BIS score.

## Method

10 patients who were scheduled for on-pump CPB were enrolled into this study and had their BIS score, age, nasopharyngeal temperature and oxygenator exhaust gas isoflurane concentration recorded at three time points chosen to represent cooling, stable hypothermia and rewarming.

## Results

The mean BIS scores was excessively low at 16 (95% CI 8 - 23), with all patients recording a mean score below the target range of 40-60. Multiple regression found a significant positive association between the temperature and BIS score (beta = 0.678, p = 0.05). No significant association with concentration of isoflurane being exhausted from the oxygenator during CPB, and temperature was found.

## Discussion/Conclusion

Administration of 2.5% isoflurane during CPB was associated with an excessive depth of anaesthesia as measured by BIS. However, the clinical importance of this finding is limited by two factors. The strong influence of body temperature on BIS and the lack of association with oxygenator exhaust concentration of isoflurane as an alternative estimate of depth of anaesthesia. If BIS is used during heart surgery then it should be interpreted with caution during hypothermic CPB.

